# A 3-Item Measure of Digital Health Care Literacy: Development and Validation Study

**DOI:** 10.2196/36043

**Published:** 2022-04-29

**Authors:** Lyndsay A Nelson, Jacquelyn S Pennings, Evan C Sommer, Filoteia Popescu, Shari L Barkin

**Affiliations:** 1 Department of Medicine Vanderbilt University Medical Center Nashville, TN United States; 2 Center for Clinical Quality and Implementation Research Vanderbilt University Medical Center Nashville, TN United States; 3 Department of Orthopaedic Surgery Vanderbilt University Medical Center Nashville, TN United States; 4 Center for Musculoskeletal Research Vanderbilt University Medical Center Nashville, TN United States; 5 Department of Pediatrics Vanderbilt University Medical Center Nashville, TN United States

**Keywords:** digital literacy, digital health care, telehealth, health equity, scale development, mobile phone

## Abstract

**Background:**

With increased reliance on digital health care, including telehealth, efficient and effective ways are needed to assess patients’ comfort and confidence with using these services.

**Objective:**

The goal of this study was to develop and validate a brief scale that assesses digital health care literacy.

**Methods:**

We first developed an item pool using existing literature and expert review. We then administered the items to participants as part of a larger study. Participants were caregivers of children receiving care at a pediatric clinic who completed a survey either on the web or over the telephone. We randomized participants into development and confirmatory samples, stratifying by language so that exploratory factor analysis and confirmatory factor analysis could be performed with separate samples of participants. We assessed the scale’s validity by examining its associations with participants’ demographics, digital access, and prior digital health care use.

**Results:**

Participants (N=508) were, on average, aged 34.7 (SD 7.7) years, and 89.4% (454/508) were women. Of the 508 participants, 280 (55.1%) preferred English as their primary language, 157 (30.9%) preferred Spanish, and 71 (14%) preferred Arabic; 228 (45%) had a high school degree or less; and 230 (45.3%) had an annual household income of <US $35,000. Using exploratory factor analysis, 3 items were retained in a reduced scale with excellent reliability (Cronbach *α*=.90) and a high variance explained (78%). The reduced scale had excellent fit, with factor loadings between 0.82 and 0.94. All fit statistics exceeded the criteria for good fit between the proposed factor structure and the data. We refer to this scale as the Digital Health Care Literacy Scale. The scale was positively associated with education (*ρ*=0.139; *P*=.005) and income (*ρ*=0.379; *P*<.001). Arabic speakers had lower scores than English (*P*<.001) and Spanish speakers (*P*=.02), and Spanish speakers had lower scores than English speakers (*P*<.001). Participants who did not own a smartphone (*P=*.13) or laptop computer (*P*<.001) had lower scores than those who owned these devices. Finally, participants who had not used digital tools, including health apps (*P*<.001) and video telehealth (*P*<.001), had lower scores than those who had used these tools.

**Conclusions:**

Despite the potential for digital health care to improve quality of life and clinical outcomes, many individuals may not have the skills to engage with and benefit from it. Moreover, these individuals may be those who already experience worse outcomes. A screening tool such as the Digital Health Care Literacy Scale could be a useful resource to identify patients who require additional assistance to use digital health services and help ensure health equity.

## Introduction

### Background

Digital technologies for managing health have proliferated in recent years, in large part as a response to the COVID-19 pandemic [1-3]. These technologies include telehealth, patient portals, and mobile health, such as app-based programs. Telehealth or the delivery of health care services at a distance using information and communication technology has been particularly helpful for allowing individuals to still receive treatment when in-person interaction is not possible. Given its convenience and now integration with many clinic workflows, it is likely that telehealth will continue to be offered as a delivery modality [4]. From a clinical trial standpoint, evidence supports the efficacy of telehealth to improve clinical outcomes across a variety of conditions [5]. Moreover, it may be more cost-effective than other approaches and patients who use it are generally satisfied with the experience [6,7]. However, to promote the continued utility of telehealth, consideration must be given to the types of individuals who have both access to, and confidence in using, it.

Despite increased access to technology over the past decade, disparities in access persist among those with lower income, with less education, or who are racial or ethnic minorities [8,9]. With respect to telehealth access, specifically, numerous studies have demonstrated stark racial and socioeconomic disparities during the COVID-19 pandemic [10-13]. This concept, known as the digital divide or the gap between people who do and do not have access to technology, affects health equity. Namely, there is concern that with the advancement in digital technologies, we may leave behind those individuals who tend to have worse health outcomes and need help the most, thereby widening the gap [14,15]. Recent efforts to expand broadband and telehealth access have begun at the federal and state levels and will require long-term, widespread investment [16,17]. An additional component of addressing the digital divide includes determining whether individuals who have access to the internet and digital devices also have the skills to use technology for accessing health care.

A certain level of digital literacy is necessary to effectively engage with, and benefit from, digital health tools. For example, individuals must feel somewhat confident in their skills using technology to install and engage with a health app or start up a telehealth visit with their provider. The telehealth modality of today’s world requires that patients initiate the visit compared with prior telehealth approaches where clinics provided all required digital health care connection. Digital literacy is considered unique and separate from technology access. In a recent study of patients admitted to the hospital, most of the participants with low health literacy had access to digital devices and had used the internet previously but were unable to perform web-based tasks without assistance [18]. Digital literacy may in fact present as a larger barrier to using digital tools than access, which underscores the need to appropriately measure and understand it.

There have been attempts in the past to measure digital literacy, although many are based on using computers generally or using the internet to find health information [19-22]. For example, the Computer Literacy Scale focuses only on computers and recognizing computer symbols and terms [22]. Likewise, the eHealth Literacy Assessment Toolkit is a compilation of scales for assessing both health literacy and digital literacy; however, the digital literacy scales primarily focus on familiarity with computer terms and confidence using computers [21]. The eHealth Literacy Scale is a popular scale for assessing electronic health literacy, but all items are anchored on using the internet to find health information [20,23]. Another scale for assessing digital literacy, the Digital Health Literacy Instrument, assesses competencies for both gathering health information and using the internet; however, the skills it assesses are very specific (ie, protecting privacy and adding self-generated content, such as writing health-related messages to a physician) [19]. It was also developed among a highly educated sample, limiting its generalizability, and the full scale consists of 21 items, limiting its efficiency [19]. To our knowledge, there are no scales that assess one’s comfort and confidence with the foundational digital skills necessary to use digital health care services such as telehealth. We refer to this as digital health care literacy. Such a scale has critical implications for clinical care and understanding the types of patients who are strong candidates for telehealth versus those who may need additional assistance.

### Objectives

To address these gaps, we sought to develop a brief measure that assesses digital health care literacy called the Digital Health Care Literacy Scale (DHLS). The item pool was developed using existing literature and expert review, and the survey was then administered to participants as part of a larger study on telehealth equity. Participants in the larger study were randomly split into development and confirmatory samples to identify a reduced version of the survey. We assessed the scale’s validity in a variety of ways, including examining its associations with digital access; prior digital health care use; and demographics, including education, income, language, race, and ethnicity.

## Methods

### Data Collection and Sample

This research was conducted as part of a larger study that sought to examine telehealth use among caregivers of young children from diverse populations. Participants were recruited from the Vanderbilt Pediatric Primary Care Clinic at Vanderbilt University Medical Center (VUMC) in Nashville, Tennessee. This clinic predominantly cares for underserved populations with higher medical and social needs. Eligible participants were aged ≥18 years; spoke a primary language of English, Spanish, or Arabic; and were a parent or guardian of a child aged <13 years who received care at the clinic between March 1, 2020, and September 30, 2020. We used the electronic medical record to query for caregivers of children who met the inclusion criteria and received permission from their providers to recruit them through telephone calls.

Interested and eligible caregivers completed informed consent and a baseline survey. All data were collected using REDCap (Research Electronic Data Capture; Vanderbilt University), a secure web-based application designed exclusively to support data capture for research studies. Surveys were administered either on the web or over the telephone and in the participant’s primary language (English, Spanish, or Arabic). Because of technical specifications in REDCap at the time of this study (ie, content was formatted to only read from left to right), all Arabic speakers (except for a single participant) completed the survey over the telephone. All key study personnel (KSP) were trained and certified before data collection and survey administration. KSP who collected survey data in a non-English language were either native speakers or fluent in the language concerned.

KSP started making telephone calls to caregivers on December 14, 2020. Rolling recruitment occurred for approximately 6 months until 500 eligible families of pediatric patients completed the survey. The final participant completed the survey on June 6, 2021. Participants were compensated with a US $15 Walmart gift card for completing the survey.

### Ethics Approval

The Vanderbilt University institutional review board approved all study procedures (approval number: 201990).

### Item Pool Development

Relevant literature and gaps in the literature were reviewed by 2 research team members (ECS and FP) to develop an initial potential item pool, covering a range of digital literacy domains and related digital skills and abilities. Input from experts from within and outside VUMC was then used to narrow down and finalize the set of items that was ultimately administered. Experts could also suggest modifications to existing items or propose new items to represent any aspects of digital literacy that may have been missing. Experts within VUMC included clinicians from the Vanderbilt Pediatric Primary Care Clinic and directors from Patient Care Operations, Interpreter Services, the Telemedicine Department, and the Department of Biomedical Informatics. We also elicited input from the manager of Nashville Public Library’s Digital Inclusion Initiatives Program. The initial item pool consisted of 81 items; after synthesizing feedback, the refined item pool for administration consisted of 6 items that were focused on confidence in the ability to use technological programs or services and the ability to independently troubleshoot technical issues (Table 1).

**Table 1:** Finalized item pool for administration and factor analysis.

Item number	Refined item pool
1	I can install applications/programs (like Zoom) on my cell phone, computer, or another electronic device on my own (without asking for help from someone else).^a^
2	I can use applications/programs (like Zoom) on my cell phone, computer, or another electronic device on my own (without asking for help from someone else).^a^
3	I can set up a video chat using my cell phone, computer, or another electronic device on my own (without asking for help from someone else).^a^
4	I can solve or figure out how to solve basic technical issues on my own (without asking for help from someone else).^a^
5	If you encounter a technical issue while using your cell phone, computer, or another electronic device, what do you do first?^b^
6	How often do you need someone (like your child/children) to help you with using your digital devices?^c^

^a^Response options range from 0 (strongly disagree) to 4 (strongly agree).

^b^Response options include 0 (ask someone for help) and 1 (try and solve the technical issue on my own—without help from someone else).

^c^Response options include 0 (always), 1 (almost always), 2 (about half the time), 3 (not very often), and 4 (never).

### Translation Process

The items were translated into Spanish by a native Spanish speaker from the VUMC Division of Academic General Pediatrics. The items were translated into Arabic by a formally trained medical translator and native Arabic speaker from the VUMC Interpreter Services Department. Both translators are fluent in, and understand, both English and their native language (Spanish or Arabic). Other than their role as translators, the Spanish and Arabic translators had no other involvement in the study. Both the Spanish and Arabic translations were checked by KSP from the research team to ensure that the translations were accurate. KSP who were involved in this process were either native speakers of, or fluent in, the language concerned. The original documents (in English), translated documents (in Spanish and Arabic), and translator declaration forms (for the Spanish and Arabic translators) were submitted to, and approved by, the Vanderbilt University institutional review board before participant recruitment and data collection.

### Measures

The survey included (1) the 6 digital health care literacy items (Table 1), (2) demographic questions, (3) questions about digital access, and (4) questions about digital health care use.

To score the digital health care literacy items (Table 1), we used a sum score of all the items such that higher scores indicated higher digital health care literacy. For item 5, we recoded the response values—0=1 and 1=3—to better align with the response values of the other items within the scale. The possible sum score for the 6 items ranged from 0 to 23.

Demographic data included age, gender, race, ethnicity, language, education, and income. Regarding digital access, participants were asked whether they owned a smartphone, a laptop computer, and/or a desktop computer. Participants were also asked about the stability of their network connection to use Internet at home and a cell phone data plan. Response options ranged from 1 (no internet or cell phone data plan) to 4 (very good). Finally, several questions assessed participants’ prior experience using digital health care. Specifically, among participants who said that they owned a smartphone, we asked whether they had ever accessed a health app. We also asked participants whether they were currently signed up for the patient portal at VUMC. The portal is a secure, web-based tool that provides patients with 24-hour access to personal health information, visit summaries, test results, and secure messaging. Finally, we asked participants whether they had used video telehealth to get care for their child in the past year. Those who had used telehealth in the past year were asked how easy it was to schedule the visit, and those who had not used telehealth in the past year were asked how difficult they thought it would be to schedule a visit. For both these items, response options ranged from 1 (very difficult) to 5 (very easy).

### Analyses

Data cleaning included evaluation of missing data, checking implausible values, and evaluating variable distributions. To evaluate the proposed scale, the participants were first divided into development and confirmatory samples so that exploratory factor analysis (EFA) and confirmatory factor analysis (CFA) could be conducted with separate samples of participants. To ensure a balanced number of surveys in each language, participants were randomized into the development and confirmatory samples stratified by language. Demographic characteristics were reported for the overall sample as well as divided by sample. Chi-square tests of independence and independent samples 2-tailed *t* tests were computed to check for balance in demographic characteristics by sample. SPSS software (version 28.0; IBM Corp) was used for data cleaning, EFA, and validation analysis. The lavaan package in R (version 3.6.2; The R Foundation for Statistical Computing) was used for CFA analysis [24], and *α* was set at .05 for statistical significance.

### EFA of Development Sample

EFA was performed on the development sample using principal axis factoring, and varimax rotation was allowed in the case of a solution with more than one factor. EFA was used to evaluate the factor structure of the 6 items proposed for inclusion in the scale as well as to reduce the 6 items to a smaller set of items that could potentially be used to measure digital health care literacy more parsimoniously. Eigenvalues >1, factor loadings >0.4, Cronbach *α*>.75, and variance explained >0.40 were used as the criteria to evaluate the items retained in the full factors and to attempt to create a reduced factor [25-27]. Once the full and reduced factors were finalized, CFA was performed on the confirmatory sample using the items suggested by the EFA.

### CFA of Confirmatory Sample

The CFA was conducted using robust maximum likelihood estimation to test the goodness of fit between the theorized factor structure suggested by the EFA and the confirmatory sample data set. The robust estimation was used because of the Likert-type ordinal responses of the items and does not assume multivariate normality of the items. A constraint value of 1 was placed on 1 item in the factor as is common in modeling analyses with a defined scale. Goodness of fit for the CFA was assessed evaluating the absolute fit, incremental fit, and parsimonious fit of the full and reduced factors [28]. The absolute fit criteria to conclude good fit between the proposed factor structure and the data included nonsignificant chi-square values, root mean square error of approximation, and standardized root mean square residual <0.08 [29]. Incremental fit criteria included the comparative fit index and nonnormed fit index >0.95. Parsimonious fit was indicated by adjusted chi-square (c2/*df*)<3.0 [30]. To assess the reliability of the full and reduced factors, Cronbach *α* was computed for the EFA and CFA. Composite reliabilities, calculated according to the weighted *Ω* formula from McDonald [31], were also calculated for the CFA because of concerns that Cronbach *α* may be inappropriate for use in structural equation modeling [32]. The variance explained was also reported for the EFA, and the average variance explained (AVE) values were calculated for the CFA with the recommended critical value >0.50 indicating that the factors explained enough of the variance in the construct [33].

### Validation

We assessed the scale’s validity by examining its associations with participants’ demographics (ie, gender, race, ethnicity, language, education, and income), digital access, and prior digital health care use. We used the Spearman *ρ* for continuous variables and the Kruskal-Wallis test for categorical variables.

## Results

### Sample Characteristics

Participants were, on average, aged 34.7 (SD 7.7) years. Of the 508 participants, 454 (89.4%) were women; 173 (34.1%) were Hispanic, 129 (25.4%) Black, 95 (18.7%) White, and 78 (15.4%) Middle Eastern; 280 (55.1%) preferred English as their primary language, 157 (30.9%) preferred Spanish, and 71 (14%) preferred Arabic; 228 (45%) had an educational attainment of high school degree or less; 230 (45.4%) had an annual household income of <US $35,000; and 351 (69.1%) had children with Medicaid insurance (Table 2). Chi-square and independent samples 2-tailed *t* tests revealed no significant differences between the development and confirmatory samples for any demographic characteristics.

**Table 2:** Demographic characteristics of the sample overall and by development and confirmatory samples (N=508).

Characteristic			Overall		Development sample (n=254)		Confirmatory sample (n=254)
Participant age (years), mean (SD)			34.7 (7.7)		34.3 (7.9)		35.0 (7.6)
Gender, female,^a^ n (%)			454 (89.4)		223 (87.8)		231 (90.9)
**Race and ethnicity,^b^ n (%)**							
	White	95 (18.7)		46 (18.1)		49 (19.3)	
	Black	129 (25.4)		64 (25.2)		65 (25.6)	
	Hispanic	173 (34.1)		88 (34.6)		85 (33.5)	
	Asian	13 (2.6)		5 (2)		8 (3.1)	
	Middle Eastern	78 (15.4)		39 (15.4)		39 (15.4)	
	Multiple	13 (2.6)		7 (2.8)		6 (2.4)	
	Prefer not to answer	7 (1.4)		5 (2)		2 (0.8)	
**Preferred language,** **n (%)**							
	English	280 (55.1)		139 (54.7)		141 (55.5)	
	Spanish	157 (30.9)		79 (31.1)		78 (30.7)	
	Arabic	71 (14)		36 (14.2)		35 (13.8)	
**Education level,** **n (%)**							
	Less than a high school graduate or GED^c^	97 (19.1)		51 (20.2)		46 (18.1)	
	High school graduate or GED	131 (25.8)		67 (26.4)		64 (25.2)	
	Some college or technical or vocational school	125 (24.6)		62 (24.4)		63 (24.8)	
	College degree (associate’s or bachelor’s)	105 (20.7)		47 (18.5)		58 (22.8)	
	Postgraduate or professional degree	49 (9.6)		26 (10.2)		23 (9.1)	
	Missing	1 (0.2)		1 (0.4)		0 (0)	
**Annual household income (US $),** **n (%)**							
	<10,000	56 (11)		33 (13)		23 (9.1)	
	10,000-19,999	45 (8.9)		26 (10.2)		19 (7.5)	
	20,000-34,999	129 (25.4)		63 (24.8)		66 (26)	
	35,000-49,999	75 (14.8)		36 (14.2)		39 (15.4)	
	≥50,000	93 (18.3)		43 (16.9)		50 (19.7)	
	Don’t know or not sure	110 (21.7)		53 (20.9)		57 (22.4)	
**Child’s or children’s insurance,** **n (%)**							
	None	7 (1.4)		3 (1.2)		4 (1.6)	
	Medicaid	351 (69.1)		184 (72.4)		167 (65.7)	
	Private insurance	75 (14.8)		33 (13)		42 (16.5)	
	Other type of insurance	74 (14.6)		34 (13.4)		40 (15.7)	
	Missing	1 (0.2)		0 (0)		1 (0.4)	
Digital Health Care Literacy Scale reduced score, mean (SD)			8.6 (3.1)		8.7 (3.0)		8.5 (3.3)

^a^Gender was assessed with the following response options: male, female, and other. No participant identified as other.

^b^Separate databases were used for English-, Spanish-, and Arabic-speaking participants, and the data were combined for analysis. Race and ethnicity were collected with a single item; the response options included White, Black, Asian, Middle Eastern, Hispanic, Native American, Native Hawaiian, other race or ethnicity, and prefer not to answer. In the English and Arabic database, participants could select all options that applied. Participants were coded as multiple if they selected more than one race and/or ethnicity, except for Middle Eastern+White, which was coded as Middle Eastern. Because of incorrect configuration, the race and ethnicity item was not enabled as a check-all item in the Spanish-speaking database (ie, these participants could only check 1 race or ethnicity). Of the 151 participants who completed the Spanish survey, 144 (95.4%) selected Hispanic, 6 (4%) selected White, and 1 (0.6%) selected Black.

^c^GED: General Educational Development.

### EFA of Development Sample

To evaluate the full digital health care literacy score, all 6 items were initially entered into the EFA, and 1 factor was extracted with an eigenvalue >1 and factor loadings between 0.45 and 0.96. Cronbach *α* was excellent at .89, and the variance explained was high at 62% (Table 3). Next, a reduced factor was created by eliminating items from the full factor one at a time and evaluating the resulting factor loadings and variance explained. Items were eliminated based on correlations with other items >0.90 with conceptual overlap (1 item; *I can install applications/programs...* overlapped with *I can use applications/programs...*) and the lowest factor loadings (2 items). On the basis of these criteria, 3 items were retained in the reduced scale (Table 3), with a resulting excellent reliability (Cronbach *α*=.90) and a high variance explained (78%).

**Table 3:** Summary of factor loadings and fit statistics for the full and reduced models.

			Full					Reduced		
			EFA^a^		CFA^b^		EFA			CFA
I can use applications/programs (such as Zoom) on my cell phone, computer, or another electronic device on my own (without asking for help from someone else)			0.96		0.95		0.96			0.94
I can set up a video chat using my cell phone, computer, or another electronic device on my own (without asking for help from someone else)			0.89		0.81		0.89			0.82
I can solve or figure out how to solve basic technical issues on my own (without asking for help from someone else)			0.81		0.84		0.80			0.85
I can install applications/programs (such as Zoom) on my cellphone, computer, or another electronic device on my own (without asking for help from someone else)			0.93		0.93		N/A^c^			N/A
If you encounter a technical issue while using your cell phone, computer, or another electronic device, what do you do first?			0.52		0.42		N/A			N/A
How often do you need someone (like your child/children) to help you with using your digital devices?			0.45		0.38		N/A			N/A
**Absolute fit**										
	Chi-square	N/A		15.99		N/A			<0.01	
	RMSEA^d^	N/A		0.07		N/A			<0.01	
	SRMR^e^	N/A		0.41		N/A			<0.01	
**Incremental fit**										
	CFI^f^	N/A		0.99		N/A			>0.99	
	NNFI^g^	N/A		0.98		N/A			>0.99	
Parsimonious fit, adjusted chi-square		N/A		1.78		N/A			0.00	
**Reliability**										
	Cronbach *α*	.89		.88		.91			.90	
	Composite reliability (coefficient *Ω*)	N/A		0.88		N/A			0.90	
	Variance explained (EFA); average variance explained (CFA)	0.62		0.62		0.78			0.75	

^a^EFA: exploratory factor analysis.

^b^CFA: confirmatory factor analysis.

^c^N/A: not applicable.

^d^RMSEA: root mean square error of approximation.

^e^SRMR: standardized root mean square residual.

^f^CFI: comparative fit index.

^g^NNFI: nonnormed fit index.

### CFA of Confirmatory Sample

Both the full and reduced factors were evaluated with CFA (Table 3). The factor loadings for the full factor ranged from 0.38 to 0.95, with model statistics of *χ*^2^_9_=16.0; *P*=.07. All the fit statistics exceeded the criteria for good fit between the proposed factor structure and the data. The reliability was excellent, with coefficient *Ω*=0.88, and the AVE was high at 0.62. The reduced scale also had excellent CFA fit, with factor loadings between 0.82 and 0.94. The chi-square value was 0, meaning the model was saturated (equal number of parameters and df). This also means that the factor was perfectly parsimonious (adjusted chi-square value of 0). All the fit statistics well exceeded the criteria for good fit between the proposed factor structure and the data. The reliability was excellent, with coefficient *Ω*=0.90, and the AVE was high at 0.75.

### DHLS Validation

#### Overview

**Figure 1:**
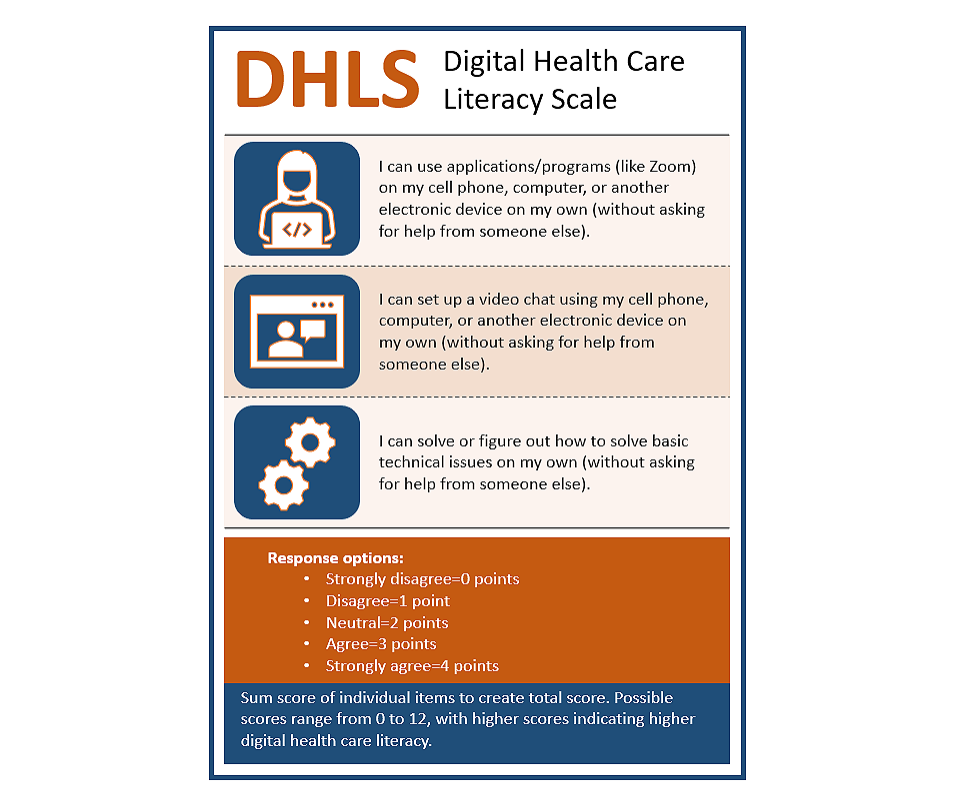
Digital Health Care Literacy Scale (DHLS).

**Figure 2:**
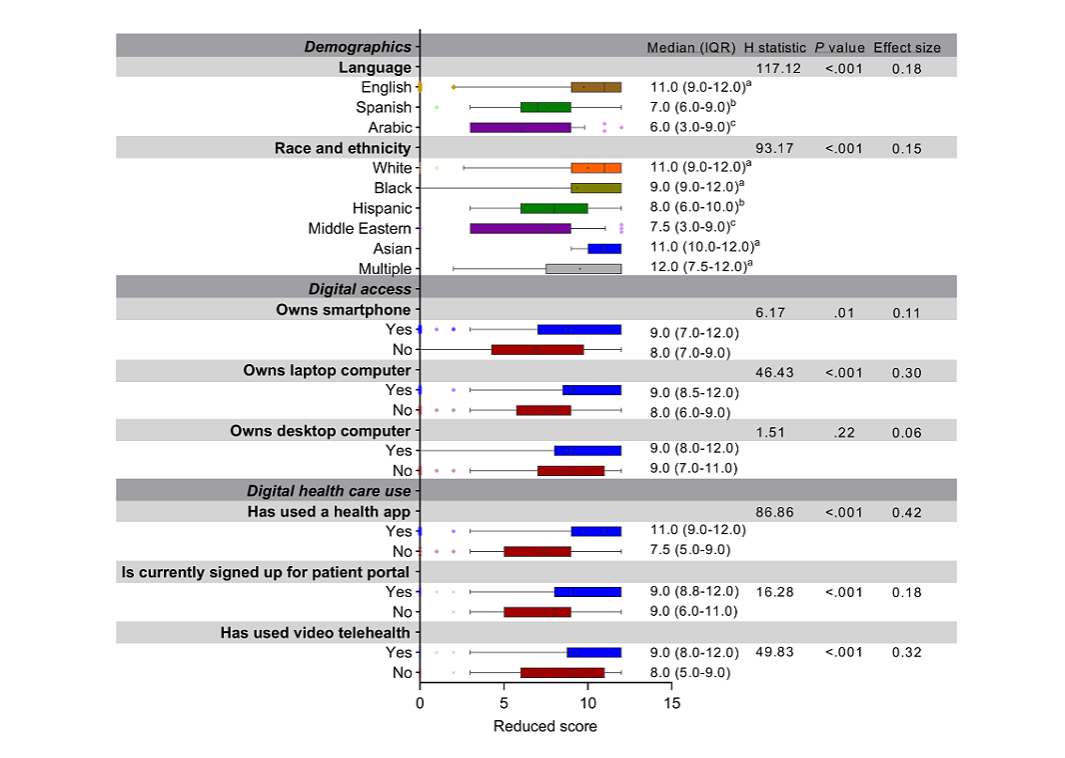
Associations between scores on the Digital Health Care Literacy Scale and participants’ demographic variables, digital access, and digital health care use. For all variables with the same superscripted designator, the difference between the medians is not statistically different. If 2 variables have different superscripted designators, they are significantly different from each other.

Because of the excellent fit of the reduced factor, we focus on this version of the scale and its validity in the following sections. Figure 1 shows the final version of the scale with response options and scoring instructions. The associations between the DHLS and measured categorical variables are shown in box plots in Figure 2. Associations with continuous variables are described only in the text.

#### Demographics

The DHLS score was negatively associated with age (*ρ*=–0.164; *P*<.001), and positively associated with both education (*ρ*=0.139; *P*=.005) and income (*ρ*=0.379; *P*<.001). There was not a significant association with gender, H_1_=1.267; *P*=.26. The overall model for language was significant, H_2_=117.115; *P*<.001. Arabic speakers had lower scores than English and Spanish speakers, and Spanish speakers had lower scores than English speakers (Figure 2). The model for race was also significant, H_5_=93.167; *P*<001. Middle Eastern participants had lower scores than all other racial groups, and Hispanic participants had lower scores than all groups, except Middle Eastern (Figure 2).

#### Digital Access

Among the 508 participants in the study, 25 (4.9%) did not own a smartphone, 191 (37.6%) did not own a laptop computer, and 401 (78.9%) did not own a desktop computer; in addition, 30 (5.9%) did not have internet access at home and 43 (8.5%) said that their internet connection was not good. We found significant associations between most of our digital access items and the DHLS score such that participants who did not have digital tool access had lower scores than those who did. Specifically, participants who did not own a smartphone or a laptop computer had lower digital literacy scores (Figure 2). However, there was not an association between desktop computer ownership and scores (Figure 2). Having a more stable network connection to use the internet at home (*ρ*=0.343; *P*<.001) and to use a cell phone data plan (*ρ*=0.312; *P*<.001) were both associated with higher scores.

#### Digital Health Care Use

Nearly half of the participants (211/508, 41.5%) had never accessed a health app, and 35.8% (182/508) were not signed up for the patient portal. Most (341/508, 67.1%) had not used video telehealth to obtain care for their children. Participants who had never used a health app had lower digital health care literacy scores than those who had (Figure 2). In addition, participants who were not signed up for the patient portal had lower scores than those who were signed up. Participants who had not used video telehealth to obtain care for their children had lower literacy scores than those who had (Figure 2). Among those who had, there was a positive association between the ease of scheduling the visit and their DHLS score (*ρ*=0.279; *P*=.001). Among those who had not used video telehealth, perceived difficulty of scheduling a visit was associated with lower scores (*ρ*=0.459; *P*<.001).

## Discussion

### Principal Findings

Given the increased reliance on digital technologies during the COVID-19 pandemic, it is critical that we understand which patients are and are not equipped for this shift in health care delivery. Without gauging patients’ confidence in skills for using telehealth and similar health care technologies, we risk exacerbating health disparities [14,17]. We developed the DHLS, a scale designed to measure an individual’s digital health care literacy, and validated it among a diverse sample of caregivers of young children. Overall, the scale had strong psychometric properties, and the reduced version of the scale performed just as well as the full version, supporting its continued and more efficient use. Participants with lower digital health care literacy had less experience with digital health care and were less likely to own digital tools. In addition, those with less education, with lower income, and people of color had lower digital health care literacy.

To our knowledge, this is one of the first tools intended to measure confidence with the skills necessary for using digital health care services, including telehealth. The Digital Health Literacy Instrument is another scale designed to measure digital health literacy; however, the items are complex and highly specific (eg, *When typing a message [e.g., to your doctor, on a forum, or on social media such as Facebook or Twitter] how easy or difficult is it for you to clearly formulate your question or health-related worry*); furthermore, the scale is long (21 items), which could lead to attrition among users with less education or literacy. The DHLS is a brief, 3-item assessment developed among a racially and socioeconomically diverse sample, and it measures the basic skills necessary for using digital health services. Of note, we focused our application of the scale in this paper on telehealth; however, it may have application to other types of digital tools. This is supported by our study, which validated the scale against the use of similar technologies (eg, whether patients had used a health app and whether they were signed up for a patient portal). Although the reduced 3-item scale is easier to administer, we encourage other researchers to use either the reduced or full scale (the latter includes additional items about digital skills, more broadly, beyond video chat) to explore other applications.

Overall, we found similar associations between participants’ characteristics and DHLS scores as other studies reporting on similar digital literacy tools. For example, having less education and lower income has previously been associated with lower eHealth Literacy Scale scores [34]. Although lower telehealth literacy was associated with older age, aligning with other studies examining digital literacy [35,36], the effect was very small. This is likely due to the limited variation in age among our sample: all participants were caregivers of children aged <13 years, with the average caregiver age being only 34.7 (SD 7.7) years. In our study, we found that Hispanic and Middle Eastern participants had lower digital health care literacy than White and Black participants, and Middle Eastern participants had significantly lower scores than Hispanic participants. A similar pattern emerged when looking at language such that Arabic speakers had the lowest digital health care literacy, followed by Spanish speakers, and then English speakers. The findings highlight the importance of examining differences in race and language by unique groups rather than collapsing groups into non-White or non-English.

Our scale could be applied as a brief assessment in clinical settings when assessing individuals’ ability to use telehealth. If a participant identifies as more digitally fluent, they may be a strong candidate for telehealth and can receive subsequent instructions for setting up a visit. However, if they identify as being less digitally fluent, resources can be provided to help that individual be better equipped for a visit. Several organizations are exploring solutions to help those with lower technology literacy prepare for telehealth appointments. For example, at VUMC, a medical student–led volunteer initiative was started to help patients set up and test devices for their telehealth appointments [37]. Students used a standardized telephone script to guide patients with downloading the proper software and understanding what to expect for the visit [37]. Another approach in Harris County, Texas, included a nonphysician staff member reaching out to ensure that patients had the proper technology and had resolved issues before the appointment [38]. Primary care practices at University of California San Francisco started an outreach program to all patients aged >65 years with scheduled visits to walk them through setting up and using the video platform app [39]. Although such initiatives have had success with preparing patients for telehealth, they are extremely time and resource intensive; a screening tool such as the DHLS could help identify only those who are most in need of assistance, thereby increasing efficiency and effectiveness. Another approach could be to simply ask patients whether they need extra help setting up a telehealth visit; however, this may have the opposite effect and lead to missing patients who do require help. That is, it is possible that some individuals may not know they need the help, especially if they have never had a telehealth visit. By using items that target the basic skills necessary to use digital tools, the scale could help to accurately identify patients who are unaware that they need assistance. Moreover, some patients may feel uncomfortable communicating that they need help. We hope that this tool provides a respectful approach for identifying those patients who require assistance.

With respect to research, the DHLS could be used as a way to help describe the digital literacy of the sample and determine whether there was representation from low digital literacy communities. It could also be useful to assess whether the use rates or efficacy of a digital technology or program were related to digital literacy. In general, we hope that the scale is included in other studies, whether for descriptive purposes, as a predictor, or as a covariate, to broaden our understanding of its applications and how it functions.

This study includes several limitations. First, these data were collected cross-sectionally; therefore, we cannot draw conclusions regarding causality. It is possible that having lower digital health care literacy leads to a lower likelihood of accessing digital health care services or vice versa. Similarly, as part of a cross-sectional study, we are limited in our ability to propose a cutoff score for determining who requires additional assistance with digital health care; however, certain study designs can effectively answer this question. For example, a future study might administer the DHLS and then attempt to conduct a telehealth visit with all participants. By examining the difference in scores between those who were and were not successful with completing the visit, we could determine a cutoff score that helps identify the likelihood of being able to successfully carry out a telehealth visit in typical circumstances. In this study, one of our goals was to explore associations between the scale and a variety of barriers to telehealth, of which scheduling a visit was one; however, scheduling a visit is likely reflecting both clinic-level and patient-level characteristics and therefore we recommend interpreting this association with some degree of caution. All participants were caregivers of children and recruited from a clinic in Middle Tennessee, which limits generalizability to other populations and other regions; however, we enrolled a racially, ethnically, and socioeconomically diverse sample of participants. We developed the items such that they can theoretically be used widely with different types of individuals, and we encourage researchers to use and validate the scale in other populations. Although the DHLS was negatively correlated with age, the sample was, on average, of younger age (mean age 34.7, SD 7.7 years), and it will be especially important to see how the scale functions with older populations who tend to experience more barriers to digital health [40-42]. In addition, although we included participants who spoke English, Spanish, and Arabic, there were likely confounding differences among the groups, and we did not use a sample-matching approach to ensure comparability of participant characteristics among languages. To consider the scale validated for all languages, a future study would need to include large numbers of individuals who spoke each language with sufficient heterogeneity and representation of participant characteristics. Relatedly, because our scale items were originally written and derived by English speakers, it is possible that the lower mean scores observed within the Spanish and Arabic groups could have been at least partially caused by intrinsic bias. Full validation within each language would help to confirm whether intrinsic bias was present. Finally, patients were not included in the development of the scale; it is possible that the inclusion of patient input could have strengthened it.

### Conclusions

Widespread adoption of telehealth by clinicians and patients alike has the potential to revolutionize health care delivery, improving both quality of life and clinical outcomes. However, as part of this quest, we must consider those patients who may not have the digital access or skills to use telehealth—in many cases, these are the same patients who tend to have worse outcomes. A screening tool such as the DHLS can be a useful resource to identify patients who require additional assistance to effectively engage with telehealth. Validating the scale among other patient populations and in other settings will support the scale’s ultimate utility to reduce health care inequities.

## Abbreviations

AVE: average variance explained
CFA: confirmatory factor analysis
DHLS: Digital Health Care Literacy Scale
EFA: exploratory factor analysis
KSP: key study personnel
REDCap: Research Electronic Data Capture
VUMC: Vanderbilt University Medical Center
